# Scaling behavior of drug transport and absorption in *in silico* cerebral capillary networks

**DOI:** 10.1371/journal.pone.0200266

**Published:** 2018-07-10

**Authors:** William Langhoff, Alexander Riggs, Peter Hinow

**Affiliations:** Department of Mathematical Sciences, University of Wisconsin - Milwaukee, Milwaukee, WI 53201-0413, United States of America; University of California Irvine, UNITED STATES

## Abstract

Drug delivery to the brain is challenging due to the presence of the blood-brain barrier. Mathematical modeling and simulation are essential tools for the deeper understanding of transport processes in the blood, across the blood-brain barrier and within the tissue. Here we present a mathematical model for drug delivery through capillary networks with increasingly complex topologies with the goal to understand the scaling behavior of model predictions on a coarse-to-fine sequence of grids. We apply our model to the delivery of L-Dopa, the primary drug used in the therapy of Parkinson’s Disease. Our model replicates observed blood flow rates and ratios between plasma and tissue concentrations. We propose an optimal network grain size for the simulation of tissue volumes of 1 cm^3^ that allows to make reliable predictions with reasonable computational costs.

## Introduction

Many diseases and conditions of the brain, such as Alzheimer’s Disease, Parkinson’s Disease (PD) and brain cancers are treated with pharmaceutical drugs. In fact, this is often the only option as the conditions are either not amenable to surgery or surgery is prohibited in this difficult terrain. A limitation to the drug treatment approach is the blood-brain barrier (BBB) that makes it highly difficult for the drug to reach its site of intended action. In its normal state the BBB protects the brain from infections and toxins. Its neurovascular unit consists of the capillary endothelial cells, astrocytes, pericytes and neurons. Because of the presence of tight junctions and efflux transporters, this structure is far less permeable than non-brain capillaries [1], although it has transmembrane carriers for the uptake of selected molecules, e.g. glucose and small molecule drugs [2]. A path to overcome these challenges to drug delivery is targeted drug delivery using nanocarriers that are loaded with drug and made to release their cargo where desired by a trigger [3–5]. Mathematical modeling and computational simulation are essential tools to further our understanding of transport phenomena in biology and biomedical engineering in general [6]. In the particular area of drug delivery, among the various topics that have been addressed are the optimization of properties of drug carrier particles [7] and the effects of physical triggers such as ultrasound [8] or elevated local temperature [9–11].

Recent work [8] proposed a compartmental mathematical model for liposomes that encapsulate a drug and release their cargo by a focused transcranial ultrasound signal. The model includes the release of the cargo from the liposomes as a function of the sound pressure, interactions of the free drug with plasma proteins [12], passive as well as nonlinear active transport mechanisms across the BBB [13] and the drug metabolism in the brain tissue [14]. The model also accounts for the temporary opening of the BBB in response to focused ultrasound [15]. However, for sake of simplicity, the model in [8] considered only a single blood vessel. Sample simulations were performed for the dopamine precursor L-Dopa and the anticancer drug doxorubicine. L-Dopa (levodopa, L-3,4-dihydroxyphenylalanine) is an amino acid of 200 Da molecular weight that crosses the BBB with the help of the LAT1 transporter [16] whereas its metabolite dopamine does not cross the BBB. L-Dopa has been used to treat motor dysfunctions associated with PD for over 40 years [17]. However, it is known that long-term L-Dopa therapy may lead to “wearing-off” symptoms such as dyskinesia and motor fluctuations. L-Dopa is currently under investigation as a candidate for targeted delivery with the goal of improving PD therapy [18]. The BBB itself has been shown to remain intact and L-Dopa transport unaffected in animal PD models [19, 20].

In this paper, we extend our previous model to a network of capillaries, however, we simplify the problem by assuming that the drug is already present in the blood. The network is modeled by a directed graph where each edge represents an individual capillary. The capillaries are characterized by their lengths and radii, and the flows through the network are determined by Kirchhoff’s Law and the Hagen-Poiseuille Equation. Similar models have been used to study the delivery and tissue transport of oxygen in the brain [21–23]. Our goal is to develop a model for drug delivery that can be adapted to specific target regions, allows to vary parameters, and offers predictions for future experimental work. The emphasis at this stage is not to reproduce the extremely complex topologies and geometries of brain capillary networks in humans or model mammals. Rather, we propose a “zero series” model that is portable for use by others. A primary goal is to keep the amount of computational complexity and the hardware requirements at a manageable level for simulations of brain tissue volumes of 1 cm^3^ and more. Though there are no principal difficulties to simulate, say, 10^7^—10^9^ ordinary differential equations on parallel computers, we feel that it is more valuable at present to understand the scaling behavior based on computations on selected networks with ≈ 10^3^ nodes. Modern imaging technology has made it possible to scan cortical portions of human brains [24–26] and to create accurate computational representations of the underlying vessel networks. However, it has to be noted that these scans were made in brains from anatomical collections and not in live patients. Furthermore, the sites at which some neurological disorders such as PD manifest are located at the geometric center of the human head, some 80-100 mm below the surface. It will be a remarkable event when in the future a living patient’s vascular network can be scanned at a depth [27] and then a computational version can be used for the simulation of a procedure.

This paper is structured as follows. We begin by describing the ordinary differential equation model for drug delivery, the construction of the capillary networks and some theoretical requirements in the definition of scaling laws. Then we present a sequence of increasingly complex networks on which we implement the differential equation model. The idea is to find a relation between predictions of the model for scenarios of different computational complexity. An integral part of this work is the simulation software which is well-documented and written in the OpenSource language python. Finally, we discuss the results of the paper in light of experimental observations of partition coefficients for L-Dopa.

## Materials and methods

### The model for drug delivery on a capillary network

**Fig 1:**
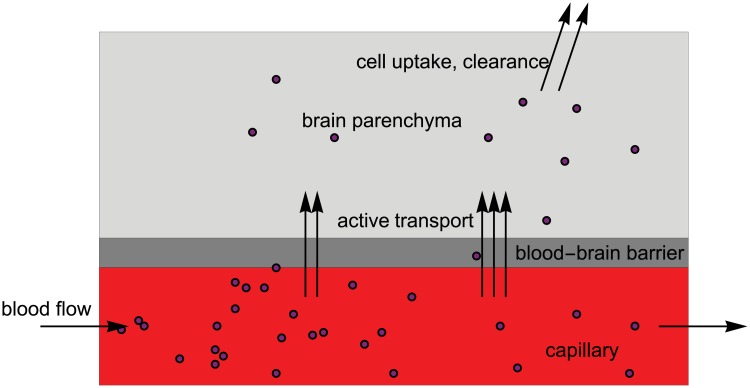
A simplified model for active drug transport within the capillaries, across the BBB and subsequent metabolism. The drug is represented by purple circles.

The process of drug delivery to the brain and transport across the BBB is described schematically in Fig 1. Here we consider only active transport across the BBB and ignore passive (Fickian) transport due to concentration differences. For sake of simplicity we also ignore binding of drug to blood plasma proteins. We create the differential equation model for a network of capillaries making the following assumptions. The construction of the underlying network is described in detail further below.

1. The capillary network has a constant topology and geometry during simulation times of interest, say, 1 day. We will work with a cubic lattice for simplicity.
2. The blood flow in the capillaries is described by a Newtonian fluid, however, the blood viscosity depends on the diameter of the capillaries (see Eq (3) below).
3. There is a constant concentration of drug in the blood entering the network.
4. Each capillary serves its own volume of brain tissue, these are mutually disjoint.
5. The drug is assumed to mix instantaneously in the tissue region associated with each capillary.

For capillary *i* = 1, 2,…,*m*, let *v*_*i*_ be the concentration of drug. The concentration of drug in the brain tissue served by this capillary is denoted by *w*_*i*_. Let *f*_*i*_ denote the reciprocal of the transit time of the blood through vessel *i* which is obtained from the volumetric flow rate, see Eq (2) below. In the following we describe the terms in each of the differential equations of the model. Let *B* denote the ratio of brain tissue volume to capillary volume for a given capillary. The transport occurs at a maximal rate *k*_1_ ≥ 0 and *K* > 0 is the drug concentration at which the transport occurs at half the maximal rate. Finally, *k*_2_ ≥ 0 denotes the rate of clearance or metabolism of the drug from the brain. The differential equations for the capillary *i* are

$$\begin{array}{c} \begin{array}{cc} \frac{dv_{i}}{dt} & =\underset{\text{loss}\;\text{to}\;\text{flow}}{\underset{︸}{-f_{i}v_{i}\left(t\right)}}\underset{\text{transport}}{\underset{︸}{-\frac{k_{1}v_{i}\left(t\right)}{K+v_{i}\left(t\right)}}}\underset{\;\text{inflow}\;\text{from}\;\text{upstream}\;\text{vessels}}{\underset{︸}{+f_{i}\frac{\sum_{j\in P_{i}}V_{j}v_{j}\left(t\right)}{\sum_{j\in P_{i}}V_{j}}}},\\ B\frac{dw_{i}}{dt} & =\underset{\text{transport}}{\underset{︸}{\frac{k_{1}v_{i}\left(t\right)}{K+v_{i}\left(t\right)}}}\underset{\text{drug}\;\text{metabolism}\;\text{and}\;\text{clearance}}{\underset{︸}{-k_{2}w_{i}\left(t\right)}}. \end{array} \end{array}$$ (1)

Here $P_{i}$ denotes the set of all parent vessels of vessel *i*, i.e. the vessels feeding directly into vessel *i*. The volumes of the parent vessels *V*_*j*_ are needed at a confluence point to ensure conservation of mass. Vessels emanating from an entrance point are supplied with a constant drug concentration *v*_0_ > 0. Given a fixed target set T, we define the combined amount of drug in the corresponding brain region by

$$W\left(t\right)=B\sum_{i\in T}V_{i}w_{i}\left(t\right).$$

We represent the capillary network as a graph where the edges stand for capillaries and the vertices are their intersections. The graph is a considerable simplification of actual vasculature since tortuous vessels are approximated by a single straight cylinder with a uniform radius, to avoid additional complications due to vessel anastomoses and tortuosity [28]. This approach also allows us to approximate blood flow by laminar flow through a fixed tube. Recall from Eq (1) that we need to know the transit time across each vessel, $f_{i}^{-1}$. To this end, we assign to each capillary in the network a radius and length and then determine the inverse of the transition time which is the volumetric flow rate *Q* divided by the vessel volume *V*,

$$f=\frac{Q}{V}.$$ (2)

We use an iterative method similar to that in [29], which has been adapted for our purposes.

For the purpose of simplicity, in this paper we work with cubic lattices that represent a tissue volume of 1 cm^3^. We designate a set of entrance and exit nodes in two opposing surfaces. We then assign a radius to each vessel from a suitable Γ-distribution. More details will be provided below. The flows through the network are determined with the help of Kirchhoff’s Current Law and the Hagen-Poiseuille Equation in analogy to Kirchhoff’s Current Law and Ohm’s Law for electrical circuits [30], respectively. Kirchhoff’s Current Law states that at any junction of edges the signed sum of flow rates is zero. For a capillary, the Hagen-Poiseuille Equation relates the length *L*, radius *r*, volumetric flow rate *Q*, and the pressure drop across the capillary by

$$\Delta P=\frac{8\mu LQ}{\pi r^{4}}.$$

Given a fixed pressure difference between the entrance node and the exit node of the network, we obtain a linear system of equations for the pressure at each internal node. From these pressures we obtain the volumetric flow rates and ultimately the transit times through every vessel. Due to the Fåhræus-Lindqvist effect, we implement a simplified dependence of the blood viscosity on the vessel diameter *d*,

$$\mu =220\text{exp}\left(-1.3d\right)+3.2-2.44\text{exp}(-0.06d^{0.645}),\;\left(\text{cP}\right)$$ (3)

at constant hematocrit 45%, see Equation (4) in [31].

### Comparisons of simulations from different networks

As stated in the Introduction, our goal is to understand the scaling of the model predictions depending on the network size. Lauwers *et al*. [25] reported numbers of 10^4^ vessel segments per mm^3^ of human brain which was largely independent of the region from which the sample was taken. This value is corroborated by the earlier results [32, 33] in mammals which have similar vascular topology. Extrapolating this information to the desired model tissue volume of 1 cm^3^ means the simulation must solve systems of 10^7^ or more ordinary differential equations. Solving systems of this size is outside the scope of the desired computational scale for this paper. Thus, we reduce the number of vessels present in the model while maintaining anatomically accurate values for the remaining morphological properties. Another quantity reported in [25] that varied only little is the volume fraction of the vessels, namely 0.5–2%, with smaller values in the deeper white matter regions. Thus in order to make simulations on different networks comparable, we enforce the constraint that the total vessel volume is the same.

The second quantity to ensure comparability of different networks is the total flow rate through the tissue volume. Physiologically, on the one hand the supply of oxygen, glucose and the removal of metabolic waste need to be maintained continuously. On the other hand, the heart creates a pressure difference in a certain range to drive this process. In actual simulations of a sequence of networks we strive to have the volumetric flow rate *Q* approach some fixed value near the expected volumetric flow rate for 1 cm^3^ of brain tissue. The pressure difference and the parameter ranges for the transport of L-Dopa across the BBB are listed in Table 1. The transport and metabolism parameters for L-Dopa were derived from sources in the literature in [8] and we use them here again.

**Table 1:** Parameter ranges governing the overall blood flow, the plasma concentration and the active transport of L-Dopa. The precise values used in each simulation are listed in the figure captions.

constant	range	references
Δ*P*	20–100 mm Hg	[23]
*v*_0_	1–10 *μ*M	[34]
*k*_1_	0.3–1.1 *μ*M s^−1^	[35–37]
*K*	30–100 *μ*M	[36, 37]
*k*_2_	3.25 ⋅ 10^−2^ s^−1^	[14]

### Sequences of lattice graphs

**Fig 2:**
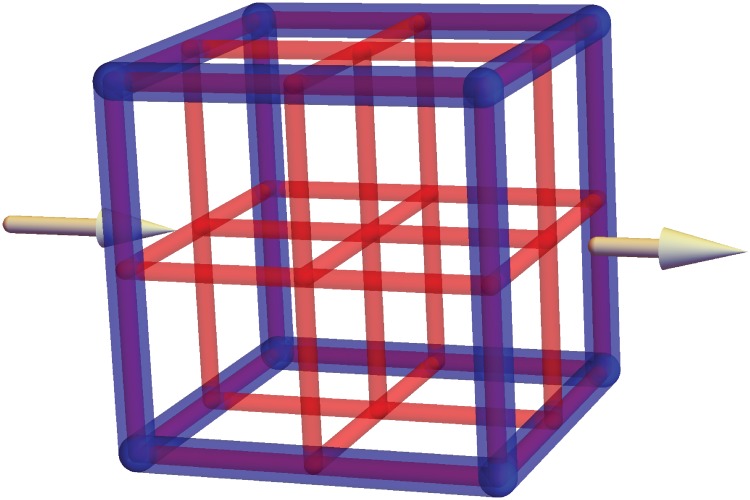
A schematic depiction of the lattice refinement process. The coarse blue network *G*_1_ is replaced by the finer red network *G*_2_. Both networks fill out the same computational volume. The arrows indicate the direction of the flow through the network.

**Fig 3:**
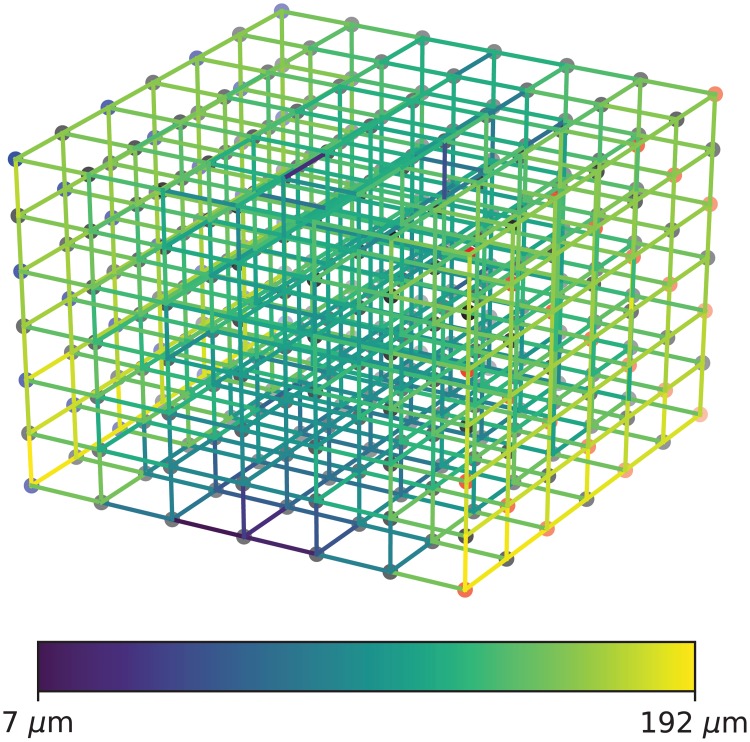
The lattice graph *G*_6_ with indicated vessel radii. The overall blood flow direction is from left to right.

We now turn to successive refinements of a cubic lattice graph which is depicted schematically in Fig 2. Each edge in the graph will be a straight vessel for the model described by Eq (1). We denote by *G*_*n*_ the graph that is obtained by subdividing the unit cube [0, 1]^3^ into *n*^3^ sub-cubes with side lengths *n*^−1^. It is easy to verify that this graph has (*n* + 1)^3^ vertices and 3*n*(*n* + 1)^2^ edges, see Fig 3. In order to obtain approximately 10^7^ edges in 1 cm^3^, we find that *n* = 149. We designate half the nodes in the surface *x* = 0 as the entrance nodes and half the nodes in the surface *x* = 1 as the exit nodes, see Fig 3. We assume that the pressure difference between the entrance and the exit nodes is constant and we ignore the pulsatile nature of the blood flow.

In order to assign a radius to each edge in the network, we fix a target tissue to vascular volume ratio of *B* = 30. For each network the radii are chosen from a Γ-distribution whose expectation *r* satisfies

$$3{\left(n+1\right)}^{2}\pi r^{2}=\frac{1}{30}.$$

For the Γ-distribution function with shape *α* and rate *β*,

$$P_{\alpha ,\beta }\left(x\right)=\frac{\beta^{\alpha }}{\Gamma (\alpha )}x^{\alpha -1}e^{-\beta x},$$

this implies that

$$r=\frac{\alpha }{\beta }=\frac{1}{\left(n+1\right)\sqrt{90\pi }}.$$ (4)

**Fig 4:**
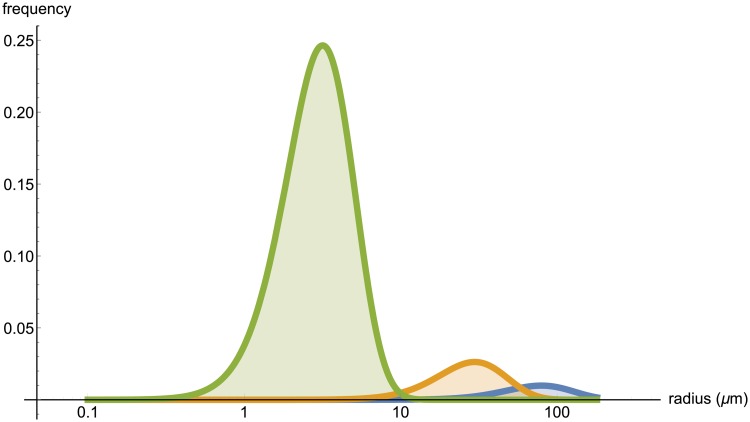
The radius distributions for the subdivision levels *n* = 5, 15 and 149 (from right to left). Note the logarithmic scale on the horizontal axis; the area under each curve is 1.

Here we have one degree of freedom and we fix *α* = 5 in order to match approximately the skewness of the distributions that were reported in Figure 5 in [24] and Figure 2A in [25]. The resulting radius distributions are drawn for selected *n* in Fig 4. For *n* = 149 we also have *r* = 3.9 *μ*m, matching the value reported by [25]. Once radii have been assigned initially at random, we enforce the constraint that those vessels further from a source or sink node will have smaller radius than those closer to a source or sink node.

## Results

The steady-state solutions of the ordinary differential equations were found with a Newton-Krylov method using the *SciPy* library for scientific computing in python [38]. All code for the numerical simulations is available on GitHub [39].

**Fig 5:**
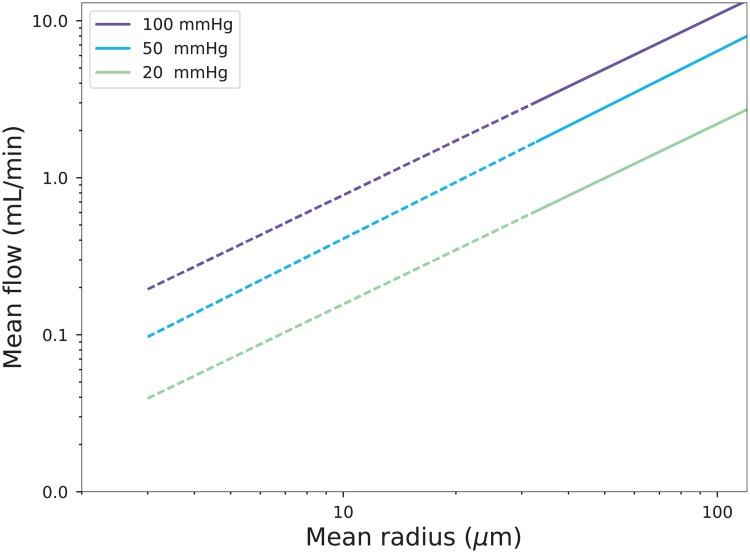
The total flow rates through several lattice graphs as a function of their average vessel radius. The dashed lines are the extrapolation from the actually computed flow rates. The legend indicates the pressure drop Δ*P*.

In Fig 5 we present the total flow rate through several networks, given different pressure differences between entrance and exit nodes. We see that for small values of the average radius *r* the extrapolated flow rate is approximately *Q* = 0.1–0.5 mL min^−1^. This is in line with the value 0.6 mL min^−1^ reported by [40] in the thalamus region. In fact, the actual flow rates may be even larger, as the blood viscosity reaches a minimum value in tubes of ≤ 10*μ*m diameter. For the following computations we fix Δ*P* = 100 mm Hg, *k*_1_ = 0.3 *μ*M s^−1^ and *K* = 101 *μ*M.

**Fig 6:**
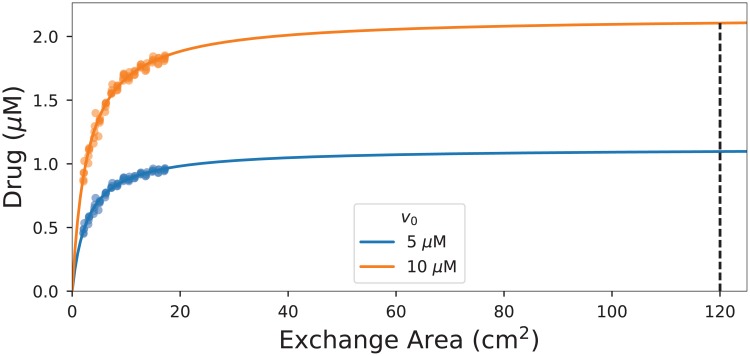
The total drug concentration at steady state as a function of the exchange area with the best fit to Eq (5). The drug concentration in the incoming blood is *v*_0_ = 5 *μ*M and *v*_0_ = 10 *μ*M, respectively. The dashed vertical indicates the extrapolated value for the estimated exchange area of 120 cm^2^.

In Fig 6 we plot the total concentration of drug in the tissue at steady state against the the total exchange area *A* of the blood vessels. The total exchange area *A* for the lattice graph *G*_*n*_ is obtained by using the average radius from Eq (4) for every one of the 3*n*(*n* + 1)^2^ vessels of length *n*^−1^. We observe a leveling off at higher exchange areas, which leads us to fit this data set to the empirical formula

$$W\left(A\right)=\frac{cA}{d+A},$$ (5)

where we use a variance weighted curve fit. As the target exchange area for our model predictions we use W* = *W*(120 cm^2)^. Note that the last simulated values are already within 88% of the extrapolated value W*. In this sense, the relatively small grid size *n* = 18 is already useful to make predictions. To give the context, the construction of the network for the last grid size *n* = 18 took one hour on a personal laptop. The steady-state computations on the same grid took 10-15 minutes.

**Fig 7:**
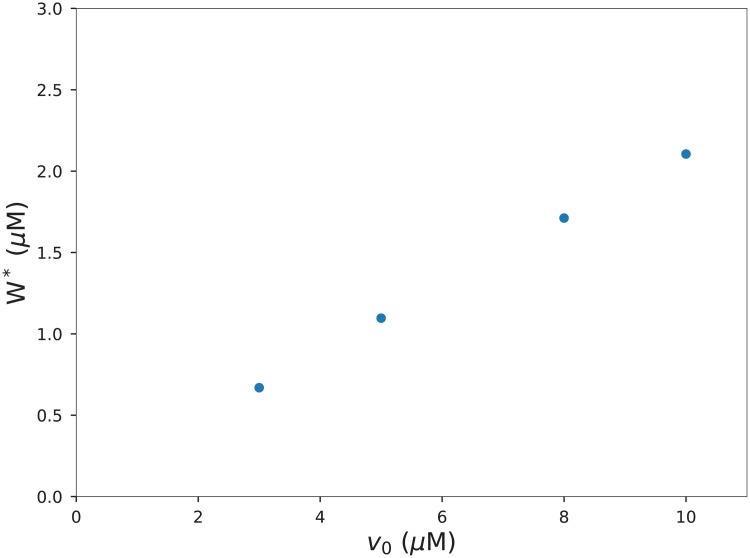
The extrapolated total drug concentration at an exchange area of 120 cm^2^ as a function of the incoming drug concentration *v*_0_.

We observe a linear relationship between the extrapolated steady state tissue concentration W* and the incoming drug concentration *v*_0_, see Fig 7. The steady state increases monotonically with the maximal transport rate *k*_1_, and decreases monotonically with the concentration at half-maximal rate of the nonlinear transporter. *K*. An increase in the metabolism or clearance rate *k*_2_ leads to a decrease in the steady-state concentration in the tissue.

**Fig 8:**
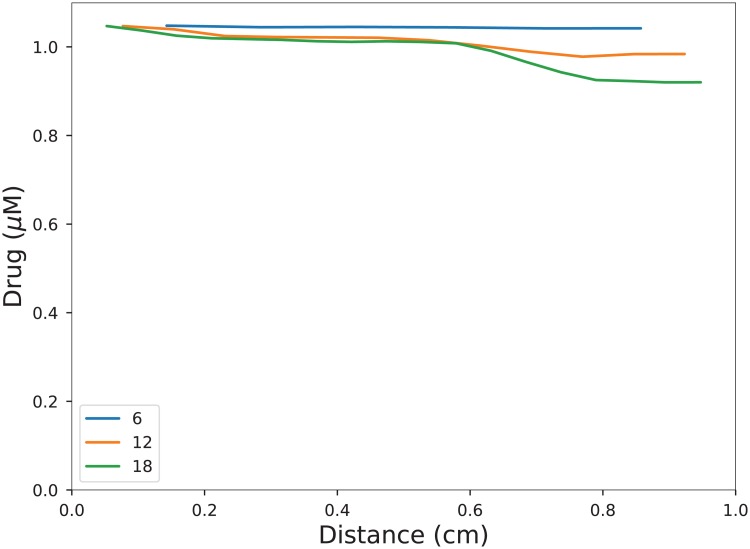
The drug concentration in the tissue as a function of the distance along the *x*-axis. The legend indicates the networks on which the steady states were found. The incoming drug concentration is *v*_0_ = 5 *μ*M.

As the blood permeates the vessel network, the drug concentration decreases, and so does the local concentration in the tissue. In Fig 8 we plot the total concentration of drug in the slices orthogonal to the *x*-direction. Taking the viewpoint from Fig 6 that the change beyond *n* = 18 is small, we propose that the last profile is close to the terminal profile. Of course, we have made the rather strong assumptions of a predominant flow direction and isolated lateral surfaces.

## Discussion

Experimental methods for probing transport across the BBB exist [41], one of the most prominent being the surgical implantation of microdialysis catheters. Using this method, [42, 43] reported that L-Dopa blood concentrations of 2 *μ*M translate into L-Dopa concentrations of approximately 0.2–0.4 *μ*M in the cerebrospinal fluid respectively in the putamen and globus pallidum interna (GPi) in patients with PD. This partition coefficient of 10–20% is in good qualitative agreement with our simulations. Note that Table 5 in [37] lists 8 values for the half-maximal transport concentration *K* of the LAT1 transporter ranging from 28.2 to 101.6 *μ*M with a mean of 60 *μ*M. These values were obtained from experiments in *Xenopus laevis* oocytes, mouse and rat cerebral epithelial cells and human intestinal epithelial cells. For the moment we will have to be content with qualitative model predictions and wider explorations of the parameter space. It is likely that for significantly smaller values of *K* diminishing returns set in at higher blood concentrations, that is, the brain concentration is a concave function of the blood concentration. It should be clarified that the whole-body pharmacokinetics of L-Dopa from stomach to brain are quite complicated and that there is a lot of variation among different patients. In addition, L-Dopa and many other drugs are administered together with auxiliary drugs whose purpose is, for example, to inhibit pathways of premature degradation [17, 42].

Several aspects of our network model deserve to be discussed. We are working with a strongly simplified network topology across all scales. Apart from simplifying the process of network construction, this also ensures greater comparability of the computational results. In future work we need to address the known changes from “tree-like” to “network-like” topology and back as the blood passes through arterioles, capillaries and venules [44]. Further, we need to allow for curved vessels [23]. The sample volume considered in this paper is significantly larger than in previously published models. Recent works have studied the influence of network topology and microvasculature morphology on computational predictions of oxygen distribution in brain tissue [45]. They concluded that the volume of modeled tissue does not affect the predicted oxygen extraction fraction and cerebral metabolic rate of oxygen.

**Fig 9:**
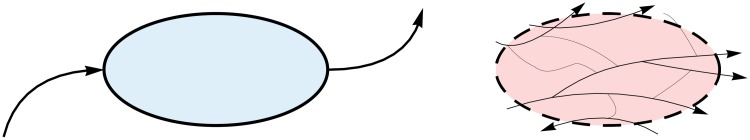
A schematic comparison of blood flow through a large organ *(left)* and an intermediate size tissue volume *(right)*.

A second difficulty, again associated with the graph depicted in Fig 3 is the presence of distinct entrance and exit points and isolated surfaces for the blood flow. Such a simplification may be defensible for the blood supply to an entire organ like the lung or the liver. A small tissue volume of a few cm^3^ within the human brain whose volume is approximately 1100 cm^3^ may have a less well-defined boundary and distributed entrance and exit points, see Fig 9. The high sensitivity of brain tissue to discontinuities in oxygen supply makes anastomoses of arteries a desirable feature [46, 47]. This raises the question how to define the pressure drop across such a region of interest and the total blood flow through it. This ambiguity affects both the computational simulation and the comparison to values for flow and pressure drop reported in the literature [23, 40]. We have been content with extrapolating a reasonable value for the flow through the network at a target mean radius *r* = 3–5 *μ*m at the upper end of the pressure interval, 100 mm Hg.

As the major transport organ of the body, blood is a natural pathway to deliver drugs to organs. In this paper we have addressed the issue of modeling the blood flow through a region intermediate in size between a few mm^3^ and the entire brain with the goal of understanding the issue of drug delivery to that region. There are computational models for the perfusion of other organs for example the liver [48] which are characterized by their own specific vascular architecture (in that case, the hepatic portal vein). Other recent works have used the mathematical technique of homogenization which leads to a limiting partial differential equation [49]. However, the nonlinear active transport of some drugs across the BBB poses a severe obstacle to this approach. While in the present work the drug is carried by the blood, in future works we will include carrier particles such as liposomes that release their cargo upon a physical trigger [5, 10]. Since the ultrasound or heat signals are choosable controls of the delivery process, the time-dependent simulations will play a more prominent role than the steady state calculations in this paper. This may also result in local drug concentrations that are temporarily much higher than the toxic systemic concentrations. These concentrations may approach saturation of the transporters, increasing the non-linear effects on the model. In future work a coupled spatial PDE model will be necessary to account for transport and diffusion of the drug after the delivery by the vessel network [50].
